# Inflammatory Markers as Predictors of Atrial Fibrillation Recurrence: Exploring the C-Reactive Protein to Albumin Ratio in Cryoablation Patients

**DOI:** 10.3390/jcm12196313

**Published:** 2023-09-30

**Authors:** Eyup Ozkan, Deniz Elcik, Suleyman Barutcu, Saban Kelesoglu, Murat Erdem Alp, Ramazan Ozan, Gazi Capar, Omer Turkmen, Goksel Cinier, Veli Polat, Mehmet Tugrul Inanc, Alper Kepez, Taylan Akgun

**Affiliations:** 1 Basaksehir Cam ve Sakura City Hospital, 34480 Istanbul, Turkey; slybrtcu@gmail.com (S.B.); muraterdemalp@gmail.com (M.E.A.); gazicapar1@gmail.com (G.C.); dromerfturkmen@gmail.com (O.T.); cinierg@gmail.com (G.C.); dr.velipolat@gmail.com (V.P.); taylanakgun@gmail.com (T.A.); 2 Faculty of Medicine, Erciyes University, 38280 Kayseri, Turkey; denizelcik@hotmail.com (D.E.); dr.s.k@hotmail.com (S.K.); ramazanozan@yandex.com (R.O.); mtinanc@msn.com (M.T.I.); 3 Department of Cardiology, School of Medicine, Marmara University, 34722 Istanbul, Turkey; alperkepez@yahoo.com

**Keywords:** atrial fibrillation, C-reactive protein, CRP albumin ratio, cryoablation

## Abstract

BACKGROUND: Atrial fibrillation (AF) is a common cardiac rhythm disorder associated with hemodynamic disruptions and thromboembolic events. While antiarrhythmic drugs are often recommended as the initial treatment, catheter ablation has emerged as a viable alternative. However, the recurrence of AF following ablation remains a challenge, and there is growing interest in exploring inflammatory markers as predictors of recurrence. METHODS: This retrospective, cross-sectional analysis included 249 patients who underwent cryoablation for paroxysmal AF. The relationship between the ‘C-reactive protein (CRP) to albumin ratio (CAR)’ and AF recurrence was examined. RESULTS: Two hundred and forty-nine patients with paroxysmal non-valvular atrial fibrillation were included. They were divided into two groups: those without recurrence (Group 1) and those with recurrence (Group 2). Significant differences were observed in age (57.2 ± 9.9 vs. 62.5 ± 8.4, *p* = 0.001) and left atrial size (4.0 ± 0.5 vs. 4.2 ± 0.7, *p* = 0.001) between the two groups. In blood parameters, significant differences were found in CRP (5.2 ± 1.3 vs. 9.4 ± 2.8, *p* < 0.001) and neutrophil counts (5.1 ± 2.2 vs. 6.7 ± 3.6, *p* = 0.001). In univariate regression analysis, age (OR: 1.058, CI: 1.024–1.093, *p* = 0.001), WBC count (OR: 1.201, CI: 1.092–1.322, *p* < 0.001), neutrophil count (OR: 1.239, CI: 1.114–1.378, *p* = 0.001), CAR (OR: 1.409, CI: 1.183–1.678, *p* < 0.001), and left atrial diameter (OR: 0.968, CI: 0.948–0.989, *p* = 0.002) showed significant associations with AF recurrence. CONCLUSIONS: Inflammation plays a crucial role in the initiation and progression of AF. This study demonstrated that along with age, the CAR can serve as an independent predictor of AF recurrence following cryoablation.

## 1. Introduction

Atrial fibrillation (AF) represents the most commonly encountered cardiac rhythm disorder in clinical practice and constitutes a critical state associated with hemodynamic disruptions and thromboembolic events [1]. Restoring sinus rhythm in appropriate patients is a significant therapeutic goal [2]. Although current guidelines often recommend antiarrhythmic drugs as the initial treatment for maintaining sinus rhythm in symptomatic patients [3], there is a growing body of evidence suggesting catheter ablation as a viable alternative for first-line therapy [4]. Previous investigations have shown that early (within 3 months) and late recurrence (within 1 year) occur in approximately 20–50% of patients following ablation for nonvalvular atrial fibrillation (NVAF) [5].

Increase in C-reactive protein (CRP) has been used as a marker of an increased inflammatory state in clinical practice [6]. An increased inflammatory state also leads to a decrease in the albumin level, which is regarded as a negative acute-phase reactant [7]. Previous studies have suggested the prognostic value of the CRP-to-albumin ratio (CAR) in conditions such as sepsis [8], cancers [9], and rheumatological diseases [10]. Evidence suggests that the CAR offers more specific results regarding inflammatory processes than CRP alone [11,12].

Inflammation plays a pivotal role in both the initiation and progression of AF [13]. It has been shown that elevated levels of proinflammatory biomarkers are associated with the development of AF [14]. Inflammation has been suggested to alter the atrial fibroblast-cardiomyocyte distribution, promoting atrial remodeling and myopathy, thereby inducing recurrence following ablation [15]. Recent studies have indicated a link between the recurrence of NVAF and the increase in new inflammatory markers such as (CRP) level, the neutrophil-to-lymphocyte ratio (NLR), and the platelet-to-lymphocyte ratio (PLR) [16,17]. A more specific marker, the CRP-to-albumin ratio, may aid in identifying patients that might have increased risk of recurrence after the catheter ablation of AF. The primary objective of this study is to examine the relationship between the CRP-to-albumin ratio and recurrence following ablation.

## 2. Materials and Methods

### 2.1. Study Population

The study was designed as a single-center, cross-sectional, and retrospective study. Study population was composed of 249 eligible patients, all over the age of 18, who had undergone cryoablation for the treatment of paroxysmal AF from May 2020 to July 2022 in ‘Basaksehir Cam ve Sakura City Hospital’, Istanbul, Turkey. Data were meticulously collected concerning their foundational clinical characteristics, in addition to preoperative echocardiographic and electrocardiographic findings. Patients presenting with persistent or longstanding persistent AF, advanced left ventricular dysfunction (left ventricular ejection fraction (LVEF) < 30%), and any previous history of AF or left atrial tachycardia ablation were excluded from the study. Other exclusion criteria were the presence of a markedly enlarged left atrial diameter (>55 mm), any inflammatory disorder, or pericarditis. Patients with a history of recent percutaneous coronary intervention or myocardial infarction (within the past three months), or a stroke or transient ischemic attack within the preceding six months, were also excluded. The study was approved by the local ethics committee, and written informed consent was obtained from all patients.

### 2.2. Laboratory Analyses

Venous blood samples were collected from the antecubital region of all patients into tripotassium ethylenediaminetetraacetic acid-based anticoagulated tubes for laboratory analysis prior to cryoablation. The samples were obtained in the morning (between 09:00 and 10:00) following a 12 h fasting period. The complete blood count, including hemoglobin, platelets, and white blood cell counts (neutrophils and lymphocytes), was measured using the Sysmex K-1000 Hematology Analyzer from Guangdong, China. High-sensitivity CRP (hs-CRP) tests and routine biochemical tests (Roche Diagnostic Gmbh, Mannheim, Germany) were performed using an autoanalyzer (Roche Diagnostic Modular Systems, Tokyo, Japan). The hs-CRP value was divided by the albumin value using units of mg/L and g/L, respectively, to calculate the CAR.

### 2.3. Follow up and Outcome

The follow-up period was a minimum of 12 months. For each patient, 12-lead ECGs were taken during the visits at 3, 6, 9, and 12 months. Patients identified with AF in these recordings were included in the recurrence group. Between routine visits, 24 h ambulatory rhythm monitoring was performed for 57 patients who complained of palpitations but had sinus rhythm on their ECGs. Among them, 35 patients with AF episodes lasting more than 30 seconds were included in the “AF recurrence” group.

### 2.4. Cryoballoon Ablation Procedure

Transthoracic echocardiographic examination was performed for all patients prior to cryobaloon ablation for the evaluation of left atrial size, left ventricular ejection fraction, left ventricular hypertrophy, and degree of mitral valve regurgitation. Transesophageal echocardiographic examination was performed within a week prior to the procedure for the evaluation of any potential left atrial thrombus. The procedure was performed under deep sedation or general anesthesia, depending on the operator’s discretion. Heparin was administered prior to transseptal puncture to maintain activated clotting time levels above 300.

LA access was obtained through a single transseptal puncture with a steerable 12 F sheath (FlexCath Advance, Medtronic, MN, USA). Following this, an Arctic Front Advance Cryoablation Catheter (Medtronic, Minneapolis, MN, USA) was guided through the FlexCath sheath to the antrum of each pulmonary vein (PV) using the Achieve Mapping Catheter (Medtronic, Minneapolis, Minnesota). For pulmonary vein isolation (PVI), the recommended protocol was to implement 240 s cryoapplications utilizing a freeze–thaw-freeze technique. This was consistent with the prevailing consensus on cryoablation application duration at the time of the trial. During the procedure, the sequence of left upper, left lower, right lower, and right upper pulmonary vein isolation was carried out. To mitigate the risk of phrenic nerve injury, right phrenic nerve pacing was conducted with an electrode catheter placed in the superior vena cava, and capture was confirmed by palpation and intermittent fluoroscopy. Cryoenergy application was ceased immediately upon any signs of diminished or lost phrenic nerve capture. Confirmation of PVI was facilitated using the Achieve mapping catheter. The electrodes of the circular mapping catheter were positioned proximal to the PV antrum for real-time PVI monitoring. The endpoint was defined as the disappearance of pulmonary vein signals on the Achieve catheter and the absence of atrial signals in the pulmonary veins, referred to as the ‘entrance block.’ If the temperature was above −55 degrees and complete PVI was determined, the procedure was finished within 180 min for each vein. If PVI was not achieved with a single freeze cycle, an additional freeze application was delivered until electrical isolation was demonstrated. All the data related with procedure were meticulously recorded in a sequential manner.

### 2.5. Medical Treatment

Post-procedurally, the patients were monitored in the ward for 24 h, and anticoagulation was initiated 4–6 h after the procedure. Systemic anticoagulation with a vitamin K antagonist (VKA) or a novel oral anticoagulant (NOAC) was recommended for at least 3 months after the procedure for all the patients. Decisions regarding the continuation of anticoagulation after 3 months were made based on the CHA2DS2-VASc risk score. Preprocedural antiarrhythmic drugs were continued for 3 months after the procedure. Decisions regarding the continuation of antiarrhythmic drugs were made based on symptoms and recurrence.

### 2.6. Statistical Analysis

All statistical analyses were performed using IBM Corp. Released 2012, IBM SPSS Statistics for Windows, version 21.0, Armonk, NY, USA: IBM Corp. software. The distribution of quantitative variables was tested using the Shapiro–Wilk test. Values displaying a normal distribution were expressed as the mean ± standard deviation, and values not displaying a normal distribution were expressed as medians with interquartile ranges. Categorical variables were expressed in ratios. Differences in continuous variables between 2 independent groups were tested using Student’s *t*-tests once normality was demonstrated. Otherwise, a nonparametric test (Mann–Whitney U test) was used. Significance of difference between categorical variables was tested using the Chi-squared test. Correlation was tested using Pearson or Spearman correlation tests where appropriate. The possible effects of different variables on AF recurrence were evaluated using univariate analysis. Variables that were found to have a significant association with AF recurrence on univariate analysis were included in the multivariate regression analysis. The predictive values of the CRP, CAR, WBC, and age were estimated by the areas under the receiver operating characteristic (ROC) curve. The area under the curve (AUC) values of each parameter mentioned were compared with the DeLong test in the MedCalc version 19.6.4, test version, statistics program (MedCalc Software Ltd., Ostend, Belgium).

## 3. Results

A total of 249 patients with paroxysmal non-valvular atrial fibrillation were included in the study. Patients were classified into two groups: those without recurrence (no recurrence group) and those with recurrence (AF recurrence group). There were no significant differences between the groups in terms of demographic data except for age (57.2 ± 9.9 vs. 62.5 ± 8.4, *p* = 0.001). There were no patients with a history of stroke in either group. Left atrium (LA) diameter was larger in patients with recurrence (4.0 ± 0.5 vs. 4.2 ± 0.7, *p* = 0.001). There was no significant difference in LVEF between the two groups. Patients with recurrence had significantly higher CRP levels (5.2 ± 1.3 vs. 9.4 ± 2.8, *p* < 0.001) and higher neutrophil counts (5.1 ± 2.2 vs. 6.7 ± 3.6, *p* = 0.001). There was no significant difference between groups related with other blood parameters (Table 1).

Univariate regression analysis revealed significant associations between AF recurrence and age [OR: 1.058, CI: 1.024–1.093, *p* = 0.001], WBC count [OR: 1.201, CI: 1.092–1.322, *p* < 0.001], neutrophil count [OR: 1.239, CI: 1.114–1.378, *p* = 0.001], CAR [OR: 1.409, CI: 1.183–1.678, *p* < 0.001], and LA diameter [OR: 0.968, CI: 0.948–0.989, *p* = 0.002]. Multivariate regression analysis revealed age [OR: 1.051, CI: 1.005–1.100, *p* = 0.029] and CAR [OR: 1.661, CI: 1.304–2.116, *p* < 0.001] as independent predictors for post-ablation AF recurrence (Table 2).

**Figure 1:**
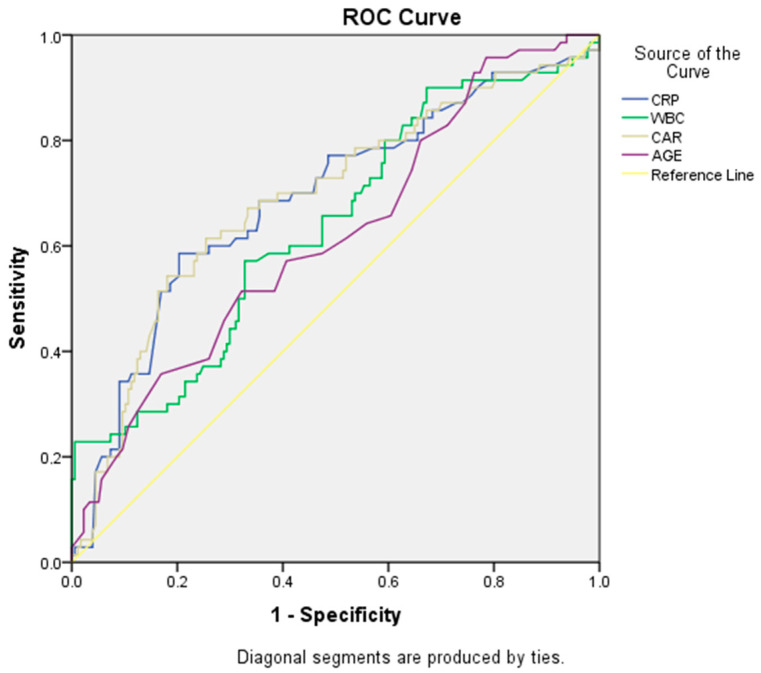
Receiver operating characteristic (ROC) curves for CRP (C-reactive protein), WBC (white blood cell), CAR (CRP albumin ratio), and age in predicting recurrence of AF. The highest AUC value was observed in ROC curve of CAR (AUC: 0.690, *p* < 0.001).

ROC curve analysis revealed that among the CRP, WBC, CAR, and age, the highest AUC curve was observed in the CAR for the prediction of post-ablation AF recurrence. A CAR value ≥ 1.11 was found to have 68% sensitivity and 67% specificity in predicting post-ablation AF recurrence (AUC: 0.690, *p* < 0.001) (Figure 1). For CRP, a value ≥ 4.5 has shown 65% sensitivity and 64% specificity in predicting post-ablation AF recurrence (AUC: 0.689). For WBC, a value ≥ 8.0 has shown 60% sensitivity and 58% specificity in predicting post-ablation AF recurrence (AUC: 0.636). For age, a value ≥ 59 years has shown 58% sensitivity and 53% specificity in predicting post-ablation AF recurrence (AUC: 0.618).

## 4. Discussion

The CAR and age were found to be independent predictors of post-cryoablation AF recurrence in this study. Other parameters significantly associated with recurrence were WBC count, neutrophil count, and left atrial diameter.

Atrial fibrillation (AF) is the most common cardiac rhythm disorder encountered in clinical practice. AF might be associated with adverse outcomes as hemodynamic perturbations and thromboembolic events [1]. Extended longevity in the general population and the increasing efforts to detect undiagnosed AF are expected to cause a 2.3-fold rise in the estimated prevalence of AF [18], which is currently between 2% and 4% in adults [2]. AF ablation has been increasingly performed in clinical practice for the restoration and maintenance of sinus rhythm in patients with AF. However, the recurrence of AF after ablation is a major challenge, with a reported probability up to 50% within 1 year after ablation [5]. Therefore, the prediction of patients who might have an increased risk of recurrence after ablation would have paramount importance. Patients without high-risk criteria for recurrence might be suggested to receive greater benefit from an invasive strategy for rhythm control. The mechanisms underlying the causes of recurrence have not yet been fully elucidated. Consequently, this ambiguity continues to pose a challenge in the management and follow-up of AF patients.

Increased age might be associated with recurrence due to a higher degree of atrial myopathy and remodeling. It can be predicted that the duration of AF will increase with age. However, age was found to be an independent risk factor for recurrence in the age-related sub-study of the CABANA trial, even in the patient groups with the same AF duration [19]. The independent association of age and AF recurrence in our study is in agreement with these observations.

Echocardiography and cardiac computed tomography are useful methods to evaluate the size and volume of the left atrium in patients with AF. Increased size and volume of the left atrium in patients with AF might be indicative of the increased fibrosis and remodeling in this chamber [20]. Decreased atrial contraction and diastolic function have been shown to be independent predictors of recurrence after ablation [21].

It is increasingly recognized that AF is not just an atrial disease; it is associated with systemic inflammation, endothelial dysfunction, cardiometabolic disturbances, and broader abnormalities in myocardial structure and function [15]. Catheter ablation is an accepted and effective treatment option for rhythm and symptom control in AF. Pulmonary vein isolation (PVI) is a gold standard procedure in AF ablation [22]. Many randomized controlled trials have compared catheter ablation to antiarrhythmic drugs in AF [23], demonstrating better efficacy and quality of life outcomes for catheter ablation [24]. Recurrence in patients after ablation is a significant clinical problem. In paroxysmal AF, the recovery of pulmonary vein conduction is the most common mechanism for AF recurrence [25]. Fibroblasts (FBs) form the cardiac skeleton, with a distribution of 70% FBs and 30% cardiomyocytes [26]. FBs stabilize the arrangement of cardiomyocytes and tissue structure by maintaining the integrity of the extracellular matrix (ECM). FBs can proliferate and differentiate into ECM-secreting cardiac myofibroblasts in response to inflammatory cytokines and other neurohumoral factors (such as transforming growth factor-B1, tumor necrosis factor-α, nuclear factor-KB) [27]. Increased ECM accumulation can lead to atrial remodeling, causing conduction abnormalities and multiple reentrant mechanisms, thus facilitating the development and persistence of AF.

Inflammation plays a role in atrial myopathy that can lead to the initiation of fibrillation by triggering an activation as powerful as pulmonary vein potentials [15]. While there are numerous studies demonstrating the relationship between inflammation and the development of AF [22], there is limited knowledge regarding the association between inflammation and AF recurrence after ablation. A previous study has suggested increased CRP levels in patients with AF [23]. The elevation was associated with the burden of AF, and patients with permanent AF had higher CRP levels compared with paroxysmal AF [23]. Both permanent and paroxysmal AF groups had higher CRP levels compared with controls in the same study [23]. We observed increased CRP levels in the AF recurrence group compared with the no recurrence group in our study. This observation might be indicative of higher baseline AF burden and higher inflammation in the AF recurrence group. However, a recently published meta-analysis reported that it was the post-ablation high-sensitivity CRP (hs-CRP) but not pre-ablation hs-CRP level that predicted AF recurrence [28]. The cause of inconsistency between our study and this meta-analysis regarding increased baseline CRP levels in patients with post-ablation recurrence is not clear, but the heterogeneity in patient characteristics might have contributed to the discrepancy. The mean age in our AF recurrent group was found to be higher compared with the group with no recurrence. It has been suggested that basal CRP levels increase with age [29]. Even though the patients included in the study had paroxysmal AF, there is evidence that the magnitude of AF burden tends to increase with advancing age [19]. Greater AF exposure may lead to increased atrial myopathy [30], which in turn may increase the inflammatory response. Larger mean left atrial diameter in the recurrent group might be suggestive of a greater inflammatory process, leading to atrial remodeling and higher baseline AF burden in these patients [31]. Based on the heterogenous results related with basal CRP levels in patients with post-ablation recurrent AF, it might be suggested that there is a need for novel markers with higher sensitivity to demonstrate the role of inflammation in the development and progression of AF. The independent association between the CAR and AF recurrence in our study might indicate that there is an increased risk in patients with a high CAR for progression to permanent AF.

Albumin is a protein produced in the liver and is a negative acute-phase reactant level which decreases in inflammatory conditions. Hypoalbuminemia has been associated with an increased incidence of various cardiovascular diseases such as ischemic heart disease, diabetes, and stroke, and a linear increase in the incidence of AF with decreasing serum albumin levels has been reported [32,33]. In addition to its association with inflammation, albumin has effects on blood viscosity, endothelial function, and platelet activation [34]. The CAR might be expected to be a more specific marker in patients with inflammatory disorders because it contains two important inflammatory biomarkers. It has been suggested that the CAR can be used as a reliable marker for increased coronary thrombus burden in patients with acute coronary syndrome [35]. The CAR has also been suggested to be a more effective prognostic marker in predicting prognosis of patients with ST elevation myocardial infarction (STEMI) compared to C-reactive protein and albumin alone [36]. There was no significant difference regarding serum albumin levels between AF recurrence and no recurrence groups in our study. Hence, it may be suggested that the difference in the CAR between the AF recurrence and no recurrence groups is largely driven by increased native CRP levels of patients in the AF recurrence group in our study.

### Limitations

The inherent limitations of a retrospective study were also valid for our study. We do not have available information which relates with the inflammatory parameters of patients after ablation. The evaluation of the trend in inflammatory markers as the CRP or CAR would have been more informative regarding the predictive role of these markers for post-ablation AF recurrence. The role of classical risk factors for AF recurrence could not be examined in detail. The information related with sleep apnea was not available for the majority of patients. The number of patients with history of thyroid disease or stroke was low, precluding detailed statistical analysis. None of these risk factors but age were correlated with recurrence in univariate analysis.

## 5. Conclusions

Inflammation has been suggested to be associated with atrial fibrillation, as with many other cardiovascular diseases. Inflammatory processes, via cytokines, cause atrial tissue remodeling and myopathy. Despite advances in catheter ablation, AF recurrence remains a significant problem, and there is limited evidence related with pathogenic mechanisms. There is a limited number of studies that have investigated the association of inflammation and AF recurrence after ablation. The CAR was found to be an independent predictor of post-ablation recurrence in our study. Based on this observation, it might be suggested that the CAR be used as a predictor of recurrence after ablation. However, the results of further large-scale prospective studies are awaited to elucidate the clinical relevance of our findings.

## Figures and Tables

**Table 1:** Baseline characteristics and operation-related data of patients with and without recurrence after AF ablation.

	No Recurrence Group	AF Recurrence Group	*p* Value
	*n* = 180	*n* = 69	
Age (year)	57.2 ± 9.9	62.5 ± 8.4	0.001
Male	88 (48%)	30 (44%)	0.401
Hypertension	74 (41%)	38 (55%)	0.102
DM	34 (18%)	16 (23%)	0.601
CAD	42 (23%)	13 (19%)	0.399
Thyroid disorder	4 (2.2%)	2 (2.8%)	0.753
Smokers	14 (7%)	6 (8%)	0.912
BMI (kg/m^2^)	24.4 ± 2.7	25.9 ± 2.3	0.463
CHA2DS2-VASc score	1.84	1.85	0.851
GFR mL/min/1.73 m^2^	94.2 ± 20.5	93.1 ± 20.5	0.756
Albumin g/L	4.3 ± 0.3	4.3 ± 0.4	0.464
Triglyceride (mg/dL)	111.3 ± 54.5	119.7 ± 68.1	0.197
LDL (mg/dL)	119.3 ± 35.4	118.6 ± 35.3	0.857
HDL (mg/dL)	36.5 ± 10.8	37.1 ± 9.1	0.553
Total cholesterol (mg/dL)	178.5 ± 45.2	185.1 ± 45.3	0.113
Serum glucose (mg/dL)	195.4 ± 89.6	183.6 ± 62.0	0.301
Hemoglobin (g/L)	14.1 ± 1.9	14.2 ± 2.0	0.933
Platelet (/mm^3^)	239.5 ± 62.5	263.7 ± 69.7	0.011
WBC (10^3^/uL)	7.8 ± 2.3	9.4 ± 3.9	0.002
Neutrophil (%)	5.1 ± 2.2	6.7 ± 3.6	0.001
Lymphocyte (%)	2.1 ± 0.9	1.9 ± 0.8	0.132
Hs-CRP mg/L	5.2 ± 1.3	9.4 ± 2.8	<0.001
CAR	1.213 (1.07–1.443)	2.238 (1.792–2.765)	<0.001
Lowest temperature achieved during ablation (°C)			
LSPV	−44 ± 4	−46 ± 2	0.322
LIPV	−45 ± 3	−44 ± 3	0.843
RSPV	−50 ± 2	−49 ± 4	0.334
RIPV	−48 ± 2	−46 ± 4	0.344
Echocardiographic findings			
LVEF (%)	53.9 ± 7.4	54.6 ± 8.7	0.248
LA diameter (cm)	4.0 ± 0.5	4.2 ± 0.7	0.001

Data are expressed as mean ± standard deviation for normally distributed data and percentage (%) for categorical variables. DM: diabetes mellitus, CAD: coronary artery disease, BMI: body mass index, GFR: glomerular filtration rate, LDL: low-density lipoprotein, HDL: high-density lipoprotein, WBC: white blood cells, Hs-CRP: high-sensitivity C-reactive protein, CAR: CRP albumin ratio, LSPV: left superior pulmonary vein, LIPV: left inferior pulmonary vein, RSPV: right superior pulmonary vein, RIPV: right inferior pulmonary vein, LVEF: left ventricular ejection fraction, LA: left atrium.

**Table 2:** Univariate and multivariate regression analyses of multiple variables on recurrence after cryoablation.

Variables	Unadjusted OR	95% CI	*p* Value	Adjusted OR	95% CI	*p* Value
**Age**	1.058	1.024–1.093	0.001	1.051	1.005–1.100	0.029
**BMI**	0.937	0.867–1.014	0.104			
**Male**	0.641	0.401–1.027	0.064			
**CAD**	0.456	0.391–1.524	0.456			
**Hypertension**	1.224	0.816–1.835	0.328			
**Smoking**	1.140	0.765–1.701	0.519			
**WBC**	1.201	1.092–1.322	<0.001	1.163	0.787–1.719	0.449
**Neutrophil**	1.239	1.114–1.378	0.001	1.069	0.709–1.611	0.751
**CAR**	1.409	1.183–1.678	<0.001	1.661	1.304–2.116	<0.001
**LA diameters on admission**	0.968	0.948–0.989	0.002	0.990	0.965–1.015	0.414

BMI: body mass index, CAD: coronary arterial disease, WBC: white blood cells, LA: left atrium, OR: odds ratio.

## Data Availability

All datasets generated during and/or analyzed during the current study are not publicly available but can be provided by the corresponding author on reasonable request.
